# miRNA-7/21/107 contribute to HBx-induced hepatocellular carcinoma progression through suppression of maspin

**DOI:** 10.18632/oncotarget.4504

**Published:** 2015-07-17

**Authors:** Wen-Shu Chen, Chia-Jui Yen, Yun-Ju Chen, Jhen-Yu Chen, Li-Yun Wang, Shu-Jun Chiu, Wen-Ling Shih, Chien-Yi Ho, Tzu-Tang Wei, Hsiao-Lin Pan, Pei-Hsuan Chien, Mien-Chie Hung, Ching-Chow Chen, Wei-Chien Huang

**Affiliations:** ^1^ Department of Pharmacology, National Taiwan University, Taipei, Taiwan; ^2^ Center for Molecular Medicine, China Medical University Hospital, Taichung, Taiwan; ^3^ Department of Family Medicine, China Medical University Hospital, Taichung, Taiwan; ^4^ Graduate Institute of Cancer Biology, China Medical University, Taichung, Taiwan; ^5^ The Ph.D. Program for Cancer Biology and Drug Discovery, China Medical University, Taichung, Taiwan; ^6^ Internal Medicine, National Cheng-Kung University, Tainan, Taiwan; ^7^ Department of Medical Research, E-Da Hospital, Kaohsiung, Taiwan; ^8^ Department of Biological Science and Technology, I-Shou University, Kaohsiung, Taiwan; ^9^ Department of Life Sciences, Tzu Chi University, Hualien, Taiwan; ^10^ Institute of Radiation Sciences, Tzu Chi Technology College, Hualien, Taiwan; ^11^ Department of Biological Science and Technology, National Pingtung University of Science and Technology, Pingtung, Taiwan; ^12^ Department of Molecular and Cellular Oncology, The University of Texas MD Anderson Cancer Center, Houston, TX, USA; ^13^ Department of Biotechnology, Asia University, Taichung, Taiwan

**Keywords:** HBx, microRNA, maspin, hepatocellular carcinoma cell, metastasis

## Abstract

Maspin suppresses tumor progression by promoting cell adhesion and apoptosis and by inhibiting cell motility. However, its role in tumorigenesis of hepatocellular carcinoma (HCC) remains unclear. The gene regulation of maspin and its relationship with HCC patient prognosis were investigated in this study. Maspin expression was specifically reduced in HBV-associated patients and correlated with their poor prognosis. Maspin downregulation in HCC cells was induced by HBx to promote their motility and resistance to anoikis and chemotherapy. HBx-dependent induction of microRNA-7, -107, and -21 was further demonstrated to directly target maspin mRNA, leading to its protein downregulation. Higher expressions of these microRNAs also correlated with maspin downregulation in HBV-associated patients, and were associated with their poor overall survival. These data not only provided new insights into the molecular mechanisms of maspin deficiency by HBx, but also indicated that downregulation of maspin by microRNAs confers HBx-mediated aggressiveness and chemoresistance in HCC.

## INTRODUCTION

Hepatocellular carcinoma (HCC), a complex and heterogeneous disease implicated by diverse risk factors, shows high invasion/metastasis and postsurgical recurrence, and low response rate to chemotherapy, leading to its poor prognosis and low survival [1, 2]. Many risk factors account for HCC, including aflatoxin, cirrhosis and hepatitis, chronic hepatitis B virus (HBV) and hepatitis C virus (HCV) infections, alcoholic liver diseases, and nonalcoholic fatty liver diseases [3]. Approximately 75% of all HCC cases are due to chronic infection with HBV or HCV viruses [4]. Chronic inflammation resulted from these viral infections disrupts the balance of damage versus regeneration in the liver, predisposing to liver cancer through fibrosis and cirrhosis stages [5]. Viral eradication of HBV and HCV has been found to efficiently reduce the risk of HCC development [6], supporting their causal role in HCC tumorigenesis. However, current curative treatment options for HCC patients are limited, and a better understanding of molecular mechanisms underlying the virus-related HCC development is important for the development of novel therapeutic approaches.

Both HBV and HCV display a strong hepatotropism, but they belong to two different viral families. HBV belongs to the family of *hepadnaviridae*, and contains a partially double stranded genomic DNA. The carcinogenic activities of HBV include insertional activation of cellular oncogene, induction of genetic instability, and modulation of host immune response [7]. HBV X protein (HBx), the smallest one of four overlapping open reading frames of HBV genome, plays pivotal roles in the tumorigenesis of HBV-associated HCC via regulating cell cycle progression, DNA repair, transcriptional regulation, signal transduction, apoptosis, and cellular adhesion [8–11]. HCV belongs to the family of *flaviviridae*, and is a single stranded RNA virus. HCV predisposes to HCC by altering cell signaling and metabolism as well as by modulating immune responses [12]. Although both chronic HBV and HCV infections are major causes of cirrhosis and HCC tumorigenesis [13], different gene expression profiles in the liver lesions of chronic hepatitis suggested that distinct pathophysiologic mechanisms may be responsible for the hepatocarcinogenesis between HBV and HCV infections [14, 15].

Mammary serine protease inhibitor (Maspin, also named serpin B5) which is a member belonging to the serine protease inhibitor (serpin) superfamily [16] has been shown to reduce tumor growth, metastasis, and angiogenesis [17–19]. It increases cellular adherence to fibronectin via inducing integrin expressions, leading to a reduction of invasion [20]. Epigenetic loss of maspin expression was observed in the advanced and chemo-refractory cancers [21, 22], supporting the anti-metastatic and pro-apoptotic properties of maspin against tumor progression and chemoresistance. However, the roles of maspin in HCC remain poorly understood. Our previous study showed an abolishment of maspin expression in HBx-overexpressing HCC cell lines [23]. Nevertheless, it is unknown whether and how HBV or HCV infection contributes to HCC progression through regulating maspin expression.

In this study, we revealed that downregulation of maspin was specifically observed in HBV-associated HCC patients and correlated with their poor prognosis. HBx is the critical regulator for suppressing maspin expression to enhance the motility and chemoresistance of HCC cells. Our data further explored that HBx induces gene expressions of microRNA-7, -21, and -107 to target the 3′-untranslational region (3′-UTR) of maspin mRNA. These results provide new insights into the molecular mechanisms of maspin deficiency in response to HBx, and suggest a positive maspin expression and low maspin-targeting microRNAs serving as biomarkers for better prognosis of HBV-associated HCC patients.

## RESULTS

### Downregulation of maspin by HBx correlates inversely with disease-free survival of HBV-associated HCC patients

**Figure 1:**
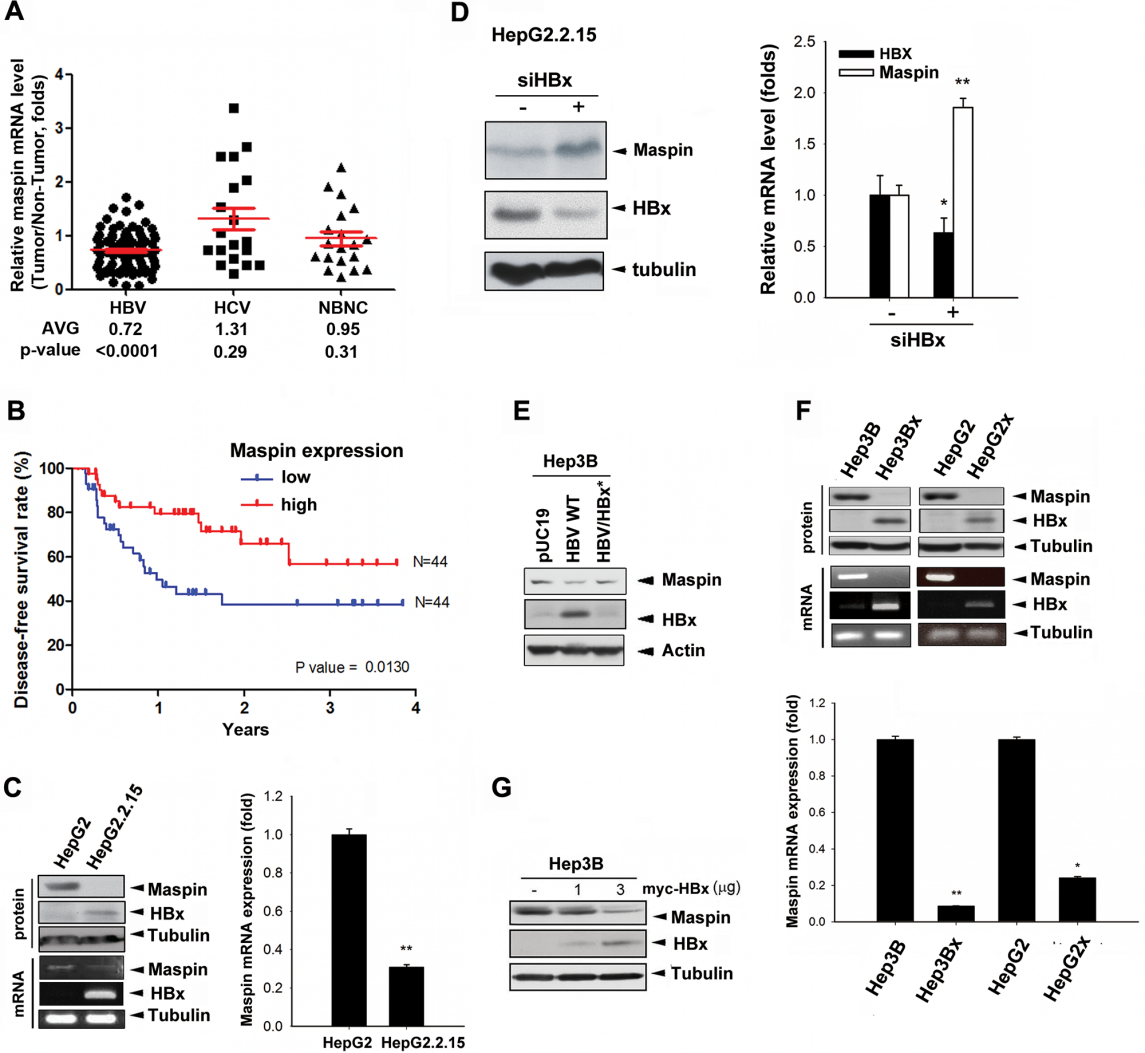
HBx-mediated maspin suppression correlated with poor prognosis of HBV-associated HCC patients **A.** Total RNA extracted from tumor and adjacent normal tissues of HBV-, HCV-, or NBNC-associated HCC patients were analyzed by real-time qPCR with maspin and GAPDH primers. The difference in maspin expression between tumor and normal tissue was presented as relative expression ratio with the normalization to GAPDH and calculated by a paired Student's *t*-test. **B.** Disease-free survival according to maspin mRNA expression level in HBV-associated HCC tissues was determined by Kaplan–Meier analysis (*n* = 88). **C–G.** Maspin protein and mRNA expression in HBV genome-(C-E) or HBx-transfected (F and G) HepG2 and Hep3B cells in the presence of HBx siRNA (*n* = 3) (D) were analyzed by Western blot, PCR, and RT-qPCR analyses. The difference was calculated by a Student's *t*-test (*p < 0.05; **p < 0.01)

The clinical relevance of maspin expression in HCC patients was first assessed. In comparison to their adjacent normal tissues, 69 of 88 HBV-associated HCC tumors (78.4%) express lower maspin mRNA level (Figure 1A) and 24 of 32 cases (75.0%) showed lower maspin protein level (Supplemental Figure S1A). The ratio of maspin mRNA and protein expression in these tumors to their adjacent normal tissue is 0.725 and 0.79, respectively. However, that was not significantly lower in HCV-associated or non-HBV and non-HCV (NBNC)-associated HCC patients (Figure 1A). The maspin expression in non-HCC liver tissue was not significantly different among groups (Supplemental Figure S1B). Importantly, disease-free survival of HBV-associated HCC patients was inversely associated with maspin expression (Figure 1B), suggesting the contribution of maspin suppression to HBV-induced HCC tumor progression. Furthermore, HBV-associated HCC patients with lower maspin expression showed a poor overall survival rate in a clear tendency to significance ( *p* = 0.0602) (Supplemental Figure S1C). The borderline significant trend is probably due to the reason that different treatments were given when HCC tumors were recurrent.

To determine the role of HBV in maspin expression, the protein and mRNA levels of maspin were examined in the stable HBV genome-transfected HepG2 (HepG2.2.15) cells, and loss of maspin protein and very low level of maspin mRNA were observed (Figure 1C). Silencing of HBx by siRNA restored the protein (left panel) and mRNA level (right panel) of maspin in HepG2.2.15 cells (Figure 1D). Consistently, transient transfection of Hep3B cells with HBV genome (HBV WT) also reduced maspin protein level, and this effect was abolished by HBx deletion (HBV/HBx*; Figure 1E). To further confirm the downregulation of maspin by HBx, HBx-overexpressing stable clones were utilized to examine its protein and mRNA levels. Maspin was diminished in HBx-overexpressing Hep3B (Hep3Bx) and HepG2 (HepG2x) cells (Figure 1F) as well as in HBx-transfected Hep3B cells (Figure 1G). Western blot analysis further showed an inverse correlation between HBx and maspin expression in a clear tendency to significance in HBV-associated HCC tumor tissues (Supplemental Figure S1D). These data indicated that HBx can downregulate maspin gene expression.

### HBx-mediated suppression of maspin contributes to metastasis, anoikis resistance, and chemoresistance

**Figure 2:**
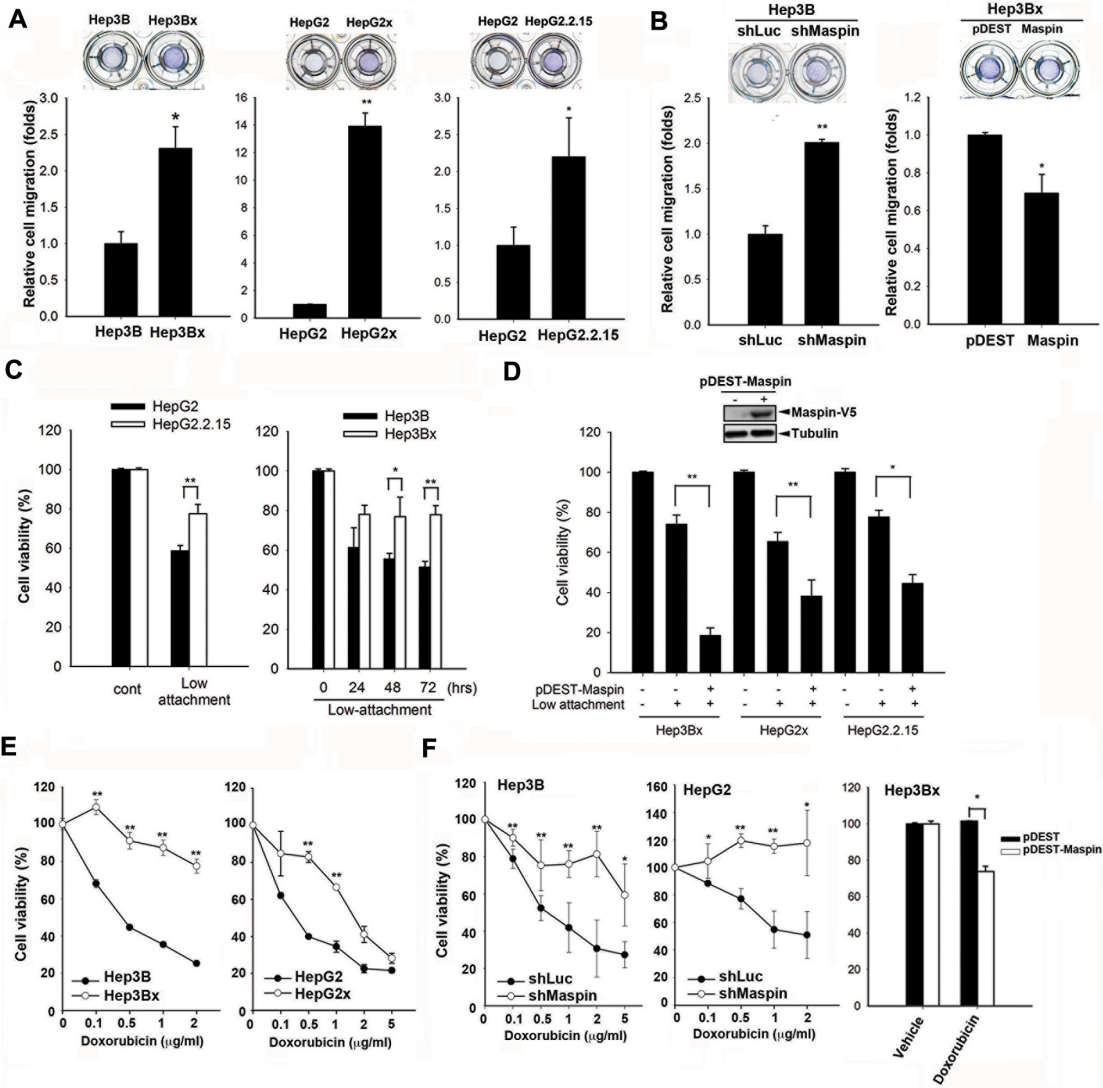
Maspin suppression confers to HBx-induced migration, anoikis resistance, and chemoresistance in HCC cells **A** and **B.** The migratory capabilities of Hep3B, Hep3Bx, HepG2, HepG2x, HepG2.2.15, and maspin-modulated cells were measured by transwell migration assay (*n* = 3). **C** and **D.** HepG2, HepG2.2.15, Hep3B, Hep3Bx, and maspin-expressing Hep3Bx, HepG2, and HepG2.2.15 cells were cultured on Ultra-low attachment plates followed by cell number counting assays (*n* > 4). **E** and **F.** Hep3B, Hep3Bx, HepG2, HepG2x, maspin-modulated Hep3B, HepG2 cells, and Hep3Bx cells were treated with indicated concentrations or 1 μg/ml of doxorubicin for 24 hours and then were subjected to MTT assay (*n* > 3). The difference was calculated by a Student's *t*-test (*p < 0.05; **p < 0.01)

To examine whether HBx-induced maspin suppression plays a role in migratory capability, transwell cell migration assays were carried out in HBx-overexpressing Hep3Bx, HepG2x, and HepG2.2.15 cells and increased cell motility was observed (Figure 2A). Silence of maspin in Hep3B cells enhanced (Figure 2B, left) but lenti-viral maspin expression in Hep3Bx cells reduced (Figure 2B, right) the cell migration, supporting that HBx increased cell migration through suppression of maspin. In addition, the enhancement of invasive ability by HBx was also observed in HBx or HBV genome-transfected HCC cells (Supplemental Figure S2A and S2B). Conversely, silence of HBx reduced the invasion ability of HepG2x cells (Supplemental Figure S2C). These results indicated that HBx also possesses the pro-invasion activity in HCC cells. Furthermore, the invasion ability was enhanced by the silence of maspin expression by siRNA, and suppressed by maspin overexpression (Supplemental Figure S2D and S2E). These findings indicated the involvement of maspin suppression in HBx-induced invasion of HCC cells.

During the process of metastasis, cancer cells detaching from extracellular matrix (ECM) evade the anoikis-induced cell death to acquire the ability to survive in circulation [25]. Therefore, the role of HBx in this event was also examined. In ultra-low attached anoikis plates, HepG2.2.15 cells bearing HBV genome showed higher viability compared to their parental cells (Figure 2C). Similarly, Hep3Bx cells and transiently HBx-transfected Hep3B and HepG2 cells were also more resistant to the anoikis-induced cell death (Figure 2C and Supplemental Figure S2F). After silencing maspin expression by shRNA or siRNA, Hep3B cells were rescued from anoikis-induced cell death (Supplemental Figures S2G). In contrast, lenti-viral overexpression of maspin increased the sensitivity of HBx-expressing HCC cells to anoikis (Figure 2D). These results support that maspin downregulation conferred HBx-mediated anoikis resistance in HCC cells.

Both HBx expression and anoikis resistance were observed to confer cancer cells resistant to chemotherapy [25–27]. The findings that Hep3Bx and HepG2x cells were more resistant to all tested chemotherapeutic agents including doxorubicin (Figure 2E), fluorouracil (5-FU), methotrexate (MTX), and paclitaxel (Supplemental Figure S3A) support the role of maspin suppression in HBx-mediated chemoresistance in HCC cells. Hep3Bx cells also showed more resistant to doxorubicin as evidenced by reduction of protein cleavages of poly ADP-ribose polymerase (PARP) and caspase-3 (Supplemental Figure S3B). Furthermore, silence of maspin by shRNA increased HCC resistance to doxorubicin (Figure 2F, left and middle) and other chemotherapeutic agents (Supplemental Figure S3C). Silence of maspin in Hep3B cells also reduced the doxorubicin-induced sub-G1 population (Supplemental Figure S3D). Conversely, overexpression of maspin can sensitize Hep3Bx cells to doxorubicin (Figure 2F, right). These results indicated that the downregulation of maspin is crucial for HBx-induced migration, anoikis resistance, and chemoresistance.

### HBx induces microRNA-7, -103, -107, and -21 to suppress maspin expression

**Figure 3:**
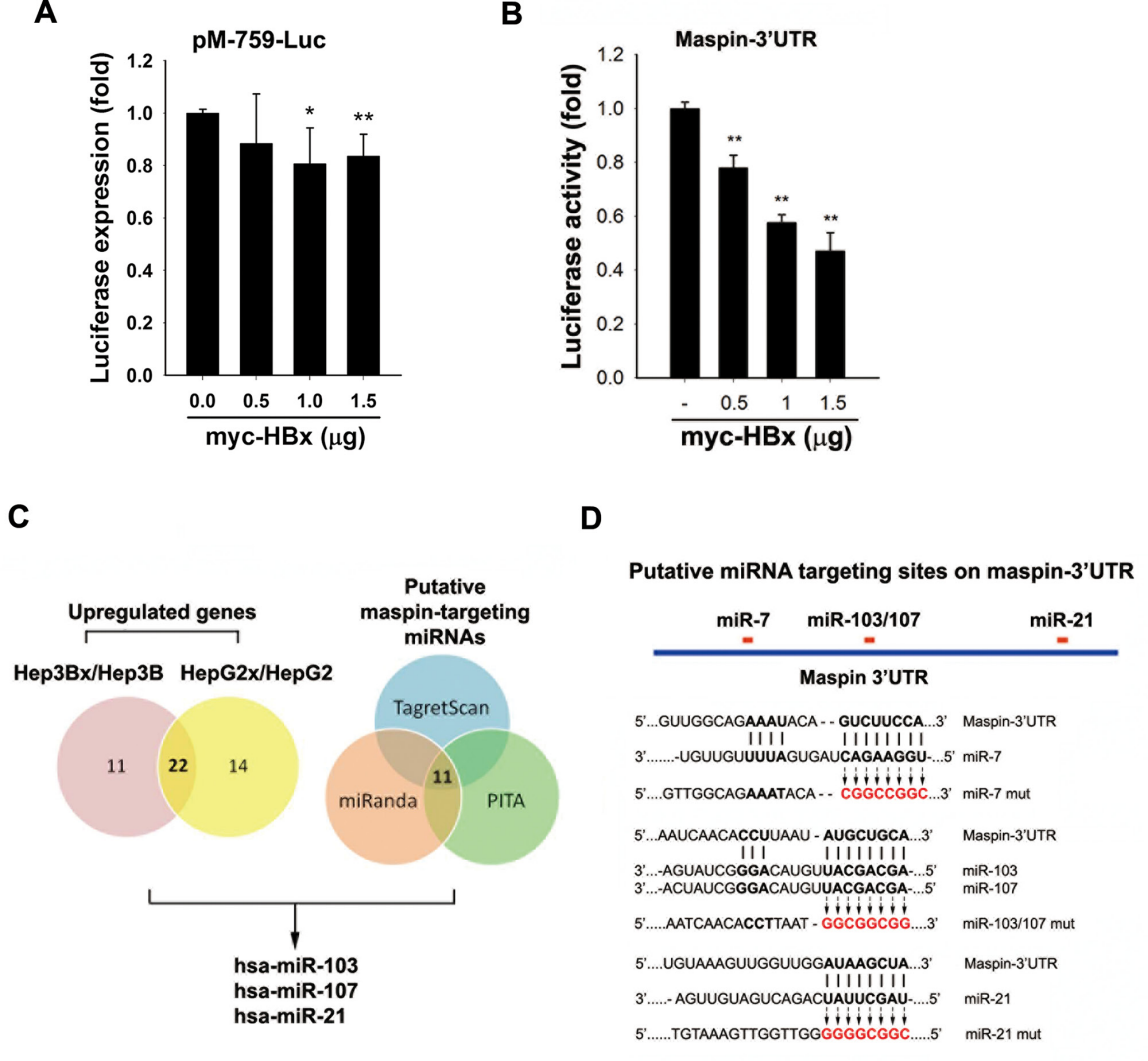
Effects of HBx on maspin promoter activity, 3′UTR activity, and maspin-targeting microRNAs induction **A** and **B.** HEK-293 cells co-transfected with maspin promoter-luciferase gene (A) or maspin 3′UTR-luciferase gene (B) and myc-tagged HBx for 24 hours were subjected to luciferase activity assays (*n* = 5 and *n* = 3, respectively). **C.** Total RNA extracted from Hep3B, Hep3Bx, HepG2, and HepG2x cells were subjected to microRNAs microarray analysis to examine the effect of HBx on the induction of microRNA expression. The putative maspin-targeted microRNAs were further analyzed by using three prediction programs. **D.** The diagram illustrates the predicted target sites of microRNAs-7, -21, and, -103/107 on maspin 3′UTR and their mutations on the maspin 3′UTR-luciferase construct. The difference was calculated by a Student's *t*-test (*p < 0.05; **p < 0.01)

**Figure 4:**
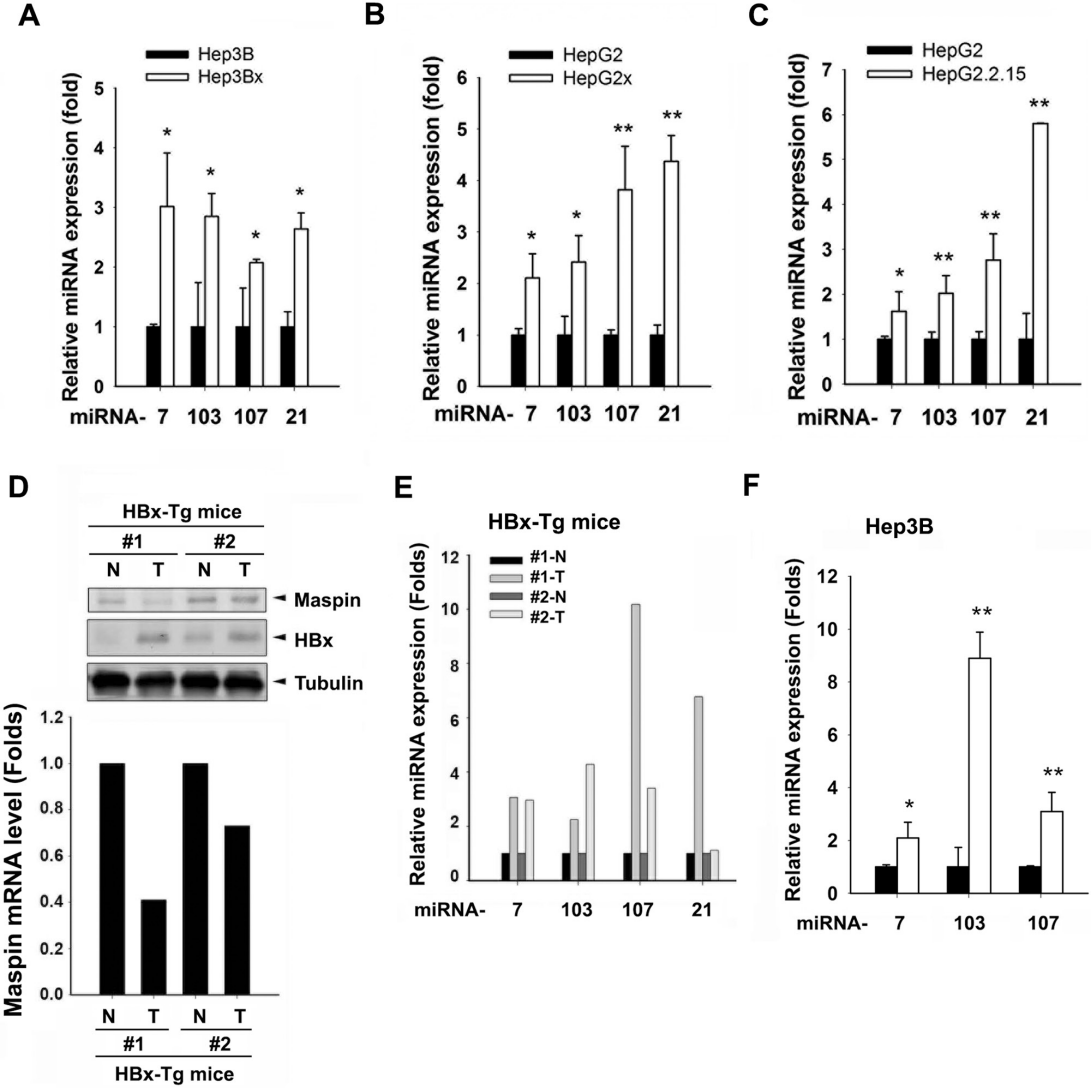
Effects of HBx on the expression of maspin mRNA, protein, and maspin-targeted miRNAs in HCC cell lines and HBx-transgenic mice **A–C.** Total RNA extracted from Hep3B, Hep3Bx, HepG2, HepG2x, and HepG2, and HepG2.2.15 cells were subjected to RT-qPCR analysis with specific primers to examine the expressions of indicated microRNAs (*n* > 4). **D-E.** Total cell lysates and RNA extracted from tumor and adjacent normal tissues of HBx-transgenic mice were analyzed by western blot and RT-qPCR. Expression levels of maspin and HBx protein, maspin mRNA, and miRNAs levels were normalized to tubulin, GAPDH, and U6B, respectively. **F.** Total RNA extracted from HBx-transfected Hep3B cells were subjected to RT-qPCR to examine the effect of HBx on the induction of microRNA expression (*n* = 3). The difference was calculated by a Student's *t*-test (*p < 0.05; **p < 0.01)

Although DNA hypermethylation has been reported to lead to maspin silence in breast cancer cells [28], transient transfection of HBx slightly reduced the maspin promoter activity (Figure 3A) but obviously inhibited the maspin 3′-UTR activity (Figure 3B). These findings suggested that HBx might downregulate maspin mRNA level through affecting its stability. Therefore, the involvement of miRNAs in maspin downregulation in response to HBx was examined by microRNA microarray analysis. There are 22 microRNAs elevated at least 2-fold in both Hep3Bx and HepG2x cells (Figure 3C and Supplemental Table S1). Among them, miR-103, -107, and -21 targeting maspin 3′UTR sequence were predicted by 3 target prediction algorithms (miRanda, Targetscan, and PITA) (Figure 3C and Supplemental Table S2). In addition, miR-7, which was observed to mediate HBx-dependent EGFR attenuation in our previous study [29], was also predicted as a maspin-targeting microRNA. Therefore, miR-7, miR-103, miR-107, and miR-21 were further investigated. The putative targeting sites of these microRNAs on maspin 3′UTR are illustrated in Figure 3D. The stable HBx-expressing Hep3Bx and HepG2x cells and stable HBV-transfected HepG2.2.15 cells (Figures 4A–4C) indeed expressed higher levels of miR-7, miR-103, miR-107, and miR-21. Consistently, liver tumor of HBx-transgenic mice also express lower level of maspin protein and mRNA (Figures 4D) but higher level of these miRNAs (Figure 4E) However, the transient HBx-transfection only induced miR-7, miR-103, and miR107 but not miR-21 in Hep3B cells (Figure 4F and data not shown), implying that HBx may immediately induce the expressions of miR-7, miR-103, and miR-107 but increase miR-21 expression after long-term expression in Hep3Bx cells.

**Figure 5:**
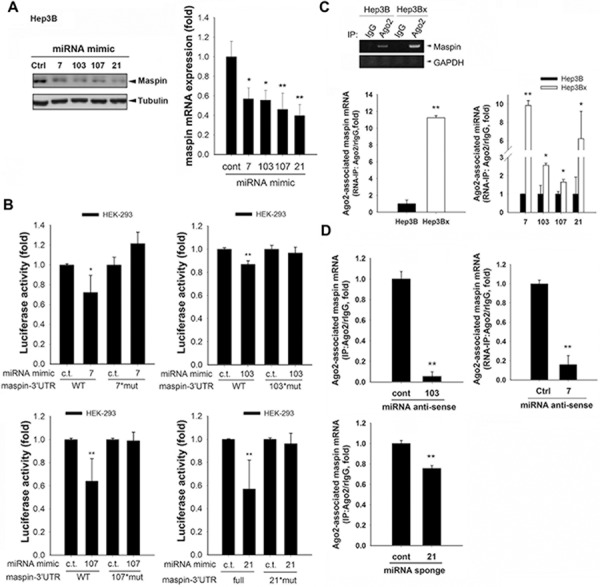
HBx-induced miRNAs suppressed maspin expression through directly interacting maspin 3′UTR **A.** Hep3B cells transfected with indicated microRNA mimics were subjected to examine the protein and mRNA levels of maspin in Western blot and RT-qPCR analyses, respectively (*n* = 10). **B.** HEK-293 cells were cotransfected with indicated microRNA mimics and luciferase constructs containing maspin 3′UTR wild type or mutated sequences for 24 hours, and were then subjected to luciferase activity assays (*n* = 5). **C.** and **D.** Hep3B and Hep3Bx cells transfected with indicated miR-7 antisense, miR-103 antisense, or miR-21 sponge were subjected to RNA immunoprecipitation with anti-Ago-2 antibody. The RNA extracted from anti-Ago2 immunoprecipitates were analyzed with maspin or indicated microRNA primers (*n* > 3). The difference was calculated by a Student's *t*-test (*p < 0.05; **p < 0.01).

**Figure 6:**
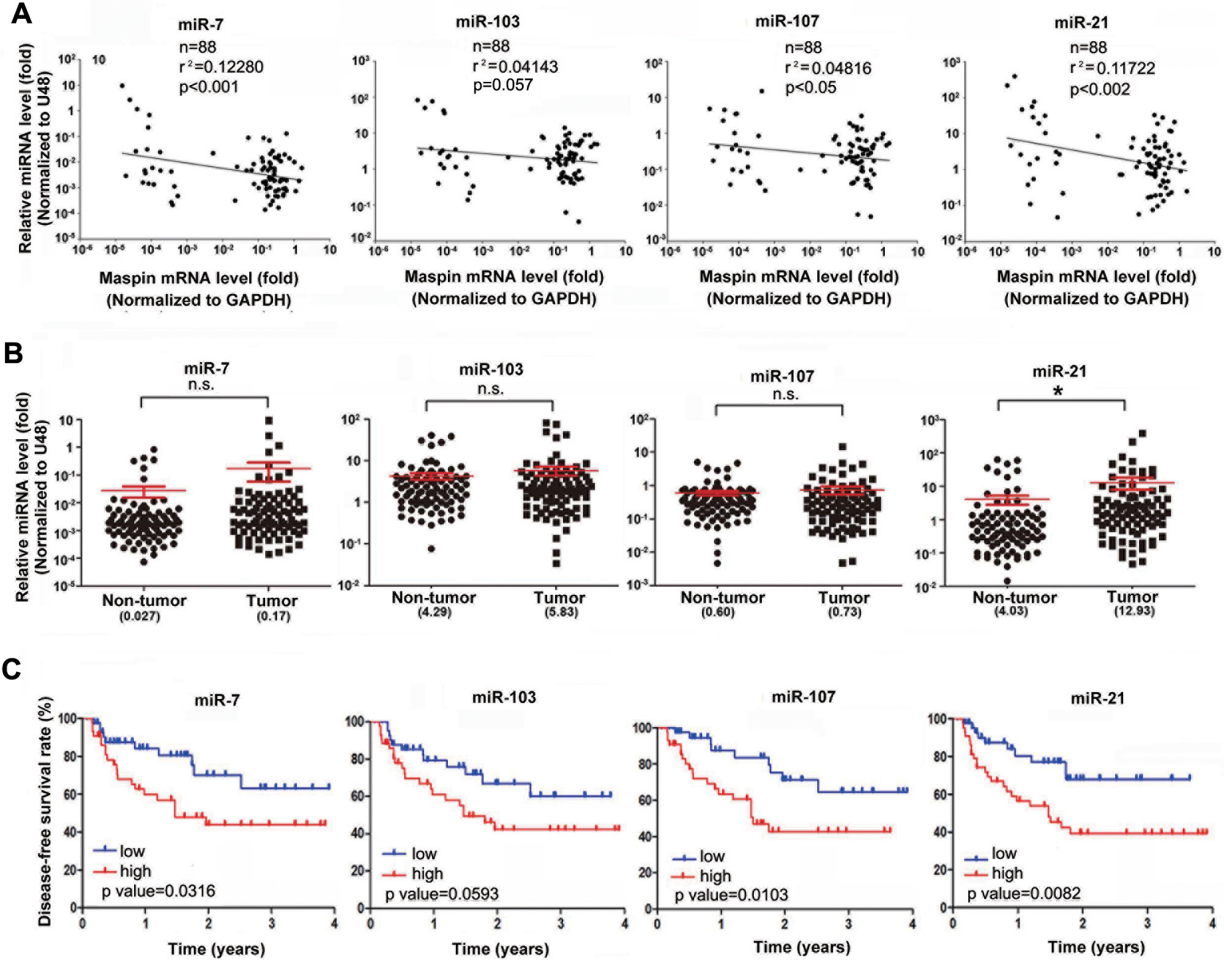
Induction of maspin-targeted microRNAs by HBx correlated with the poor prognosis of HBV-associated HCC patients **A–C.** The total RNA extracted from tumor and adjacent normal tissues of 88 HBV-associated HCC patients was analyzed by RT-qPCR for the 2^(−ΔCt) levels of indicated microRNAs with the normalization to U48 and maspin with the normalization to GAPDH (*n* = 88). The coefficient of determination (r^2^) between individual miRNA and maspin expression levels was analyzed by a coefficient analysis (A) The differences of these microRNA expressions in HCC tumor tissues compared to their adjacent normal tissues were determined in the two-tailed paired Student's *t*-test (B) Disease-free survival according to indicated miRNA levels in HBV-associated HCC tissues was determined using the Kaplan–Meier methods (C) (*p < 0.05; **p < 0.01; ***p < 0.001)

To further support the role of these miRNAs in dowrengulating maspin, mimics of miRNA-7, -103, -107, and -21 were utilized and were observed to remarkably reduce the protein and mRNA expressions of maspin (Figure 5A) as well as the luciferase activity of mapsin-3′UTR (Figure 5B). The putative binding sequences on maspin 3′UTR were mutated to identify the target sites of these microRNAs as illustrated in Figure 3D. As predicted, specific mutations of individual miRNA targeting sequences prevent the inhibition on maspin 3′UTR activity (Figures 5B). Argonaute (Ago) proteins are the catalytic components of the RNA-induced silencing complex (RISC) responsible for miRNA-mediated gene silencing [30]. The maspin mRNA detected in anti-Ago2 immunoprecipitates was higher in Hep3Bx cells than in Hep3B cells, and the Ago2-associated miR-7, miR-103, miR-107, and miR-21 were also increased in response to HBx overexpression (Figure 5C). The antisense oligonuecleotide against miR103, miR7, and miR21 reduced the Ago2-associated maspin mRNA in Hep3Bx cells (Figure 5D), supporting the direct targeting of maspin 3′-UTR by these microRNAs. Importantly, the inversed correlation of maspin expression with miR-7, miR-107, and miR-21 levels reached statistical significance and also showed clear tendency to significance with miR-103 level (Figure 6A). In comparison to non-tumor parts, the expressions of these microRNAs were higher in HBV-associated but not in HCV-associated or non-HBV/HCV HCC tumors, and the increase in miR-21 expression reached the statistical significance (Figure 6B and Supplemental Figure S4, respectively). The expression of miR-21 was also significantly higher in HBV-associated HCC tumors than in HCV-associated or non-HBV/HCV HCC tumors (Supplemental Figure S5), suggesting that these microRNAs mediate maspin suppression and miR-21 is the most critical regulator in HBV-associated HCC tumors. Moreover, the higher levels of miR-7, -107, and -21 were significantly associated with a poor disease-free survival of HBV-associated patients. However, the association of higher expression of miR-103 with the survival rate only showed a clear tendency to significance (*p* = 0.059 in Kaplan-Meier survival test and *p* = 0.06 in Cox proportional hazards model analysis) (Figure 6C and Supplemental Table S3). These data suggested that HBx-induced miR-7, miR-107, and miR-21 suppress maspin expression by directly targeting its mRNA 3′UTR and thereby confer the poor prognosis in HBV-associated HCC patients.

## DISCUSSION

Maspin is ubiquitously expressed in multiple normal tissues and epithelial cells but is downregulated in tumor cells [31]. Although the reduced expression of maspin in HCC cell lines compared with normal hepatocytes has been reported [32], the role of maspin in HCC tumor progression remains unclear. In this study, our data showed that lower expression of maspin is associated with poor prognosis of HBV-associated HCC patients, suggesting that maspin is a favorable prognostic biomarker for such patients. We further demonstrated that HBx-mediated maspin downregulation contributed to tumor metastasis, anoikis resistance, and chemoresistance in HCC cells. Although HBV and HCV infections are both major causes of HCC, the distinct expression patterns suggest the different molecular mechanisms in HCC development [14, 15]. Maspin downregulation was mainly observed in HBV-associated but not in HCV- or non-HBV/non-HCV-associated HCC patients (Figure 1A), and transfection of HBV genome but not its HBx-deletion mutant suppressed maspin expression (Figure 1E), implying the involvement maspin suppression specifically in HBx-mediated HCC tumorigenesis.

Although loss of maspin expression was also frequently occurred in advanced and chemo-refractory cancers [22, 28, 33], doxorubicin resistance led by overexpression of maspin via inducing collagen-enriched microenvironment was observed in breast and ovarian cancer cells [34], revealing the opposing functions of maspin in different cancer types. Our data showed that the HBx-mediated maspin downregulation not only increases the migration and invasion abilities but also confers anoikis resistance and chemoresistance of HCC cells. Our data further showed that expression of claudin-1, a regulator of tight junction for inducing EMT, and BCRP, an ABC transporter mediating chemoresistance, were inversely correlated with maspin expression in HBx- or HBV-stable clones of Hep3B or HepG2 cells (Supplemental Figure S6A). Restoration of maspin suppressed the expressions of caludin-1 and BCRP (Supplemental Figure S6B), suggesting that HBx-induced maspin loss may contribute to migration and chemoresistance through regulating these membrane proteins. In addition, expression of maspin reduces metastasis of breast cancer cells via repressing urokinase plasminogen activator (uPA)/urokinase plasminogen activator receptor (uPAR) complex, which enhances the cleavage of a number of extracellular matrix proteins through activating plasminogen [35]. HBx-positive HCC cells were frequently observed to express urokinase plasminogen activator (uPA) [36], suggesting that HBx-induced maspin repression may promote metastasis through elevation of uPA. HBx protein has also been indicated to induce the expression of histone deacetylase 1 (HDAC1) in HCC cells [37]. Interestingly, the inhibition of HDAC1 by maspin has been proposed to abrogate the epigenetic silence of Bax and p21^WAF1/CIP1^ gene expressions [38]. Maspin was also reported to increase the acetylation of DNA repair protein Ku70, which causes its dissociation from Bax and leads to apoptosis [39]. These observations suggest that HBx-induced maspin suppression might render cancer cells more insensitive to chemotherapy through inhibition of pro-apoptotic gene expressions or leasing Bax protein from mitochondria by Ku70 protein via increasing HDAC1 function. However, the detailed mechanisms await further investigations.

Aberrant cytosine methylation and chromatin condensation of maspin promoter have been reported to cause the transcriptional silence of maspin expression [21, 28]. However, our study provided further evidences that HBx reduced maspin expression through microRNA-dependent downregulation rather than transcriptional suppression. Among them, miR-21 showed the most inhibition efficacy and substitution of its putative binding site on maspin 3′UTR also showed the most inhibition on the 3′UTR luciferase activity (Figure 5A and 5B). Consistently, the expression of miR-21 is higher in HBV-associated HCC tumors even compared with HCV- or NBNC- tumors patients and revealed the most significant correlation to maspin expression and disease-free survival rate. These data suggested that miR-21 showed a higher targeting efficiency to mediate maspin suppression and might be a more reliable marker to predict HCC patients' prognosis.

Nuclear IKKα-mediated histone H3 Ser10 phosphorylation has been proposed to suppress maspin transcription in prostate cancer cells [22]. Nevertheless, H3 Ser-10 phosphorylation is demonstrated to promote gene transcription by increasing subsequent histone acetylations [40], suggesting that H3 Ser10 phosphorylation by nuclear IKKα may also downregulate maspin expression indirectly through induction of its negative regulators, such as microRNAs. Our previous study has demonstrated that HBx induced nuclear IKKα translocation through Akt-dependent Thr-23 phosphorylation to promote motility of hepatocarcinoma cells [23]. Moreover, our unpublished data further found that overexpression of IKKα can increase the expressions of microRNA-7, -103, and -107. These observations imply the critical regulatory role of nuclear IKKα in HBx-mediated microRNA induction and maspin suppression. However, the detailed mechanisms await further investigations.

Taken together, our study demonstrated that HBx enhanced the levels of microRNA-7, -107, and -21 to promote HCC tumor progression involving migration, invasion, anoikis resistance, and chemoresistance by directly targeting and suppressing maspin expression. Low expression of maspin and high levels of microRNA-7, -107, and -21 were strongly associated with the poor survival of HBV-related HCC patients. This study not only provides the molecular insight into the maspin suppression in response to HBx, but also suggests that these microRNA and maspin expressions in combination are potential biomarkers for the prediction of HCC patient survival.

## MATERIALS AND METHODS

### Cell culture

Human embryonic kidney 293 (HEK293) cell line and human hepatocellular carcinoma cells Hep3B, Hep3Bx, HepG2, and HepG2x were obtained from Mien-Chie Hung, Ph.D. (The University of Texas, M. D. Anderson Cancer Center, Houston). The HBx-overexpressed stable clones, Hep3Bx and HepG2x, were established from Hep3B and HepG2, respectively, and selected with G418 antibiotic agent. HepG2 and HepG2.2.15 human hepatocellular carcinoma cells were kindly provided by Wen-Ling Shih, Ph.D. (Department of Biological Science and Technology, National Pingtung University of Science and Technology, Taiwan). The HepG2.2.15 cells was established by transfecting the HBV genome into HepG2 cells and selecting with G418. All cells were cultured in Dulbecco's modified Eagle's medium/F12 medium containing 10% fetal bovine serum, 100 U/mL penicillin G, and 100 mg/mL streptomycin sulfate, at 37°C humidified incubator under an atmosphere of 5% CO2 in air. Cells were daily checked by morphology and tested to be Mycoplasma free by DAPI staining within the last 6 months.

### Microarray analysis

Microarray procedures were performed as previously described (Fukuda et al., 2007). Briefly, total RNAs from Hep3B, Hep3Bx, HepG2, and HepG2x cells were isolated using the mirVana™ RNA Isolation kit (Ambion) according to the manufacturer's protocol. A flashPAGE™ fractionator System (Ambion) was used to enrich miRNA from total RNA. One hundred micrograms of total RNA was enriched for small RNA species, tailed with the mirVana™ miRNA Labeling Kit (Ambion), and fluorescently labeled through use of the CyDye Mono-Reactive Dye Pack (GE Healthcare Bio-Science Corp). Unincorporated dyes were removed with a second glass fiber filter-based cleaning procedure. Hybridization was carried out on DNA oligonucleotide probes from the mirVana™ miRNA Bioarray V9.2 (Ambion). Following hybridization, the miRNA arrays were scanned using a GenePix 4000B scanner (Axon Instruments). Raw data were normalized and analyzed using Array-Pro Analyzer Version 4.5 (Media Cybernetics, Inc.). The data have been deposited in NCBI's Gene Expression Omnibus and are accessible through GEO Series accession number GSE56424 [24].

### Clinical specimens

HCC tissue sections and specimens were purchased from Taiwan Liver Cancer Network, Zhunan, Taiwan and provided from National Cheng Kung University Hospital, Tainan, Taiwan. Informed consents were signed by patients with approval by the Institutional Review Board, China Medical University Hospital, Taichung, Taiwan (DMR101-IRB1-119) and by the Institutional Review Board of the Human Investigation Committee of College of Medicine, National Cheng Kung University Tainan, Taiwan (B-ER-102-210). The clinical sample information met REMARK (REporting recommendations for tumor MARKer prognostic studies) guideline was shown in the Supplemental Figure S7. One hundred and seven patients who had primary HCC underwent hepatectomy at National Cheng Kung University Hospital from January 2007 through December 2012, were included. Patients with a previous diagnosis of cancer, positive surgical margins and a diagnosis of combined hepatocellular-cholangiocarcinoma were excluded. Twelve patients were excluded due to a positive surgical margin and seven patients were excluded due to a diagnosis of combined hepatocellular-cholangiocarcinoma. The remaining eighty-eight patients were included for further study (Supplemental Figure S7 and Supplemental Table S3). The follow-up interval was every 3 months. Recurrence of HCC was documented upon typical findings of computed tomography or magnetic resonance imaging with or without raised serum AFP level or pathological confirmation. Disease-free survival (DFS) was defined as time from surgery to the first occurrence of either local or distant recurrence. Disease-specific survival (DSS) was defined as time from surgery to HCC-related death. Subjects were censored at the last follow-up appointment or at death without recurrence. The patient profiles of the 88 HCC patients were shown in Supplemental Table S3. The patients included 52 (59.1%) males and 36 (40.9%) females with age range of 42 to 78 years (mean age 59.7 years). The median follow-up time was 28 months (range, 3 to 46.5 months). At the end of the follow-up, thirty-eight patients (46.6%) had recurrent HCC (median duration until recurrence, 9 months; range, 2.5–38.6 months), including local recurrence in 28 patients, metastasis in 6 patients, and both local recurrence and metastasis in 4 patients. The 1-year disease specific survival rate was 91.4% and 3-year disease specific survival rate was 65.6%. Informed consent in writing was obtained from each patient and the study protocol conformed to the ethical guidelines of the 1975 Declaration of Helsinki as reflected in a priori approval by the Human Experiment and Ethics Committee of National Cheng Kung University Hospital in Tainan, Taiwan. The diagnosis of HBV and HCV-associated HCC subtype was made on the basis of presence of HBV or HCV surface antigen. The characteristics of these patients were described in Supplemental tables S3 and S4. Expressions of Maspin mRNA (Figure 1A) and microRNAs (Figure 6) normalized respectively to GAPDH and U48 were determined in RT-qPCR analysis. Their differences in various HCC tumor tissues compared to the normal tissues was determined in the paired two-tailed Student's *t*-test and *p* < 0.05 was defined as statistically significant.

### Anoikis assay

Anoikis assay was performed with cell culture on Ultra-Low attachment Costar 24-well plates (Corning, NY) for 48 hrs. After induction of anoikis, the cell viability were calculated with trypan blue staining and counted by using Countess Automated Cell Counter (Invitrogen, Carlsbad, CA).

### Preparation and infection of shRNA-, antisense-miR-103-, antisense-miR-7 and sponge-miR-21- expressing lentivirus

Briefly, 2 μg pCMV-dR8.91, 200 ng pMD2.G, and 2 μg pLKO-shLuciferase, pLKO-shmaspin, antisense-miR-103, antisense-miR-7 or sponge-miR-21 were cotransfected into HEK293T cells using Lipofectamine 2000. The supernatants containing infectious lentivirus were collected after 1 day of transfection. For lentivirus infection, cells (2 × 10^5^) were infected with lentivirus at a multiplicity of infection (MOI). After 5 days infection, cells were harvested for protein or RNA extraction.

### RNA immunoprecipitation

Briefly, cells were crosslinked with formaldehyde for 15 min and then treated with Glycine for 5 min. After wash with cold PBS, cells were lysed in 1 ml of lysis buffer followed by vortex. The lysates were cleared by centrifugation, and protein concentrations were assessed by BCA kit (Thermo, Lafayette, CO, USA). For immunoprecipitation, lysate was incubated with anti-Ago2 antibody-coated Sepharose beads overnight at 4°C. After wash with cold NT2 buffer and incubation with proteinase K (10 mg/ml) at 55°C for 30 min, co-immunoprecipitated RNA was extracted with Tripure isolation reagent, and then subjected to RT-qPCR.

### Statistical analysis

The difference in relative gene expression between tumor and normal tissues was calculated by a two-tailed Student's *t*-test. Coefficient analyses were performed for the correlation between gene expressions. The percentage of cumulative survival was determined by Kaplan-Meier survival test. The univariate and multivariate analyses were used in Cox proportional hazards models. All these statistical analyses were performed using Sigma Plot 10.0. A *p*-value < 0.05 was defined as statistically significant.

Other experimental procedures were described in Supplemental Materials and Methods.

## SUPPLEMENTARY DATA FIGURES AND TABLES


