# Supplementary material for: miRNA-7/21/107 contribute to HBx-induced hepatocellular carcinoma progression through suppression of maspin
Source: Oncotarget. 2015 Jul 17;6(28):25962–74. doi: 10.18632/oncotarget.4504 (PMC4694878; doi:10.18632/oncotarget.4504)
Supplement: Supplementary file 1 [file oncotarget-06-25962-s001.pdf]

SUPPLEMENTARY DATA

Plasmids, antibodies, and chemicals

pLenti6/V5-DEST-maspin plasmid was constructed using Gateway® technology (Invitrogen, Boston, USA) according to the manufacturer's instructions. Maspin-3′UTR was constructed in to pMIR-REPORT™ Luciferase plasmid (Ambion, Austin, TX, USA) flanked with HindIII and XhoI cutting sites. Mutations of indicated sites in maspin-3′UTR were generated using a Quickchange site-directed mutagenesis kit (Stratagene, La Jolla, CA, USA) according to the manufacturer's instructions. All of the above plasmids were confirmed by DNA sequencing. We purchased antibodies against maspin from Santa Cruz (Santa Cruz, CA, USA), antibodies against ERK1/2, and agonaute 2 from Cell Signaling Technology (Beverly, MA, USA), and antibody against HBx from Abcam (Cambridge, UK). The validated siRNA for negative control, maspin, miRNA mimics, and miRNA inhibitors were all purchased from Dharmacon (Lafayette, CO, USA). The validated shRNA for negative control and maspin were purchased from National RNAi Core Facility at Academia Sinica (Taipei, Taiwan). The sponge empty control and miR21 were purchased from addgene. The lentiviral expression vector expressing hairpin sequence against miR-103–1, scramble control, and pPACKH1™ Packaging Plasmid mix were purchased from SBI and produced lentivirus according to the manufacturer's instructions (System Biosciences, miRZip, San Francisco, CA, USA). Tripure isolation reagent for RNA isolation was purchased from Roche (Indianapolis, IN, USA). The MMLV First-Strand cDNA Synthesis kit and Universal probelibrary Probe#21 was purchased from Roche (Indianapolis, IN, USA). The KAPA SYBR® FAST Master Mix (2X) and KAPA Probe FAST Universal qPCR Kit were purchased from Kapa biosystem (Woburn, MA). The chemotherapeutic drugs, doxorubicin hydrochloride, vinorelbine ditartrate salt hydrate, 5-fluorouracil, methotrexate, and paclitaxel were all purchased from Sigma-Aldrich (St. Louis, MO, USA). Luciferase assay system was purchased from Promega (Madison, WI, USA).

Transient transfection

Cells at 60% confluence were transfected with indicated plasmids or siRNA using Nanofectin (PAA, Pasching, Austria), Lipofetamine 2000 (Invitrogen, Boston, USA), or DharmaFECT (Thermo Scientific Dharmacon, Lafayette, CO, USA) according to manufacturer's instruction. Nanofectin and Lipofectamine 2000 were used in plasmid DNA transfection, and the DharmaFECT was used in siRNA transfection. Briefly, plasmid DNA or siRNA were mixed with transfection reagents with 1:2 ratio in serum-free DMEM medium at room temperature for 30 min or 1 hour and then were added into culture medium for 6 h at 37°C followed by refreshment with complete DMEM medium. After 48 h, cells were subjected to total lysate preparation, total RNA extraction, MTT, cell cycle analysis, or transwell migration assays.

Protein extraction and immunoblot

For total cell lysates, cells were washed with ice-cold PBS one time and lysed in RIPA buffer (20 mM Tris-HCl, pH7.4, 150 mM NaCl, 1% NP-40, 1% sodium deoxycholate, 1 mM EDTA and 1 mM EGTA) containing protease inhibitors and phosphatase inhibitors cocktails (Roche, Indianapolis, IN, USA). Proteins were separated by SDS-PAGE, transferred to PVDF membrane, and blotted with indicated antibodies.

Reporter gene luciferase assay

The luciferase reporter gene pM-Luc(-759) plasmid containing promoter of maspin was a gift from Prof. Shiv Srivastava (Center for Prostate Disease Research, Uniformed Services University of the Health Sciences). Cells with 60–80% of confluence were transfected with maspin-3′UTR or pM-Luc(-759) luciferase plasmids along with or without miRNA mimics, miRNA inhibitors, or myc-HBx expression vectors. After 48 hrs of transfection, cell lysates were harvested and subjected to luciferase assay system. Luciferase activity was normalized to β-gal activity.

3-(4, 5-dimethylthiazol-2-yl)-2, 5-diphenyltetrazolium bromide (MTT) cell viability assay

In vitro cell viability was measured using an MTT colorimetric assay. Cells (5×10^3 to 1×10^4 cells/well) were seed in 96-well plate. After treatment, the culture medium was removed and 1 µg/ml MTT solution (Sigma, St. Louis, MO, USA) was added to incubate for 3 hours. Finally, DMSO was added to lyse the cells and the absorbance at OD_{550} wavelength was detected by ELISA reader.

Migration and invasion assays

Transwell migration and invasion assays were carried out using transwell chambers (24-well insert; pore size, 8 mm; Costar Corp., Cambridge, MA). For invasion assays, a thin layer of Matrigel was coated onto a porous membrane in transwell chambers. Cells (2×10^5 to 4×10^5) were seeded on the non-coated membrane of the upper chamber. After incubation for 48 h, cells remained inside the upper chamber were removed with cotton swab. Cells migrated or invaded through the pores to the opposite side

of the membrane were fixed with methanol and stained with crystal violet, followed by microscopic observation. The migratory cells were then lyses with 33% (v/v) acetic acid solution and quantified by absorbance measurement (OD_{570}).

Semi-quantitative reverse transcription-polymerase chain reaction (RT-PCR) and Quantitative real-time PCR (RT-qPCR)

Total RNA was extracted by using Tripure isolation reagent according to the manufacture's instruction. One µg of RNA was subjected to reverse transcription with the MMLV First-Strand cDNA Synthesis kit. After reverse transcription, PCR was carried out for amplification of maspin, HBx, and GAPDH using Prime Taq DNA polymerase (Genet Bio, Korea). The amplified products were visualized on a DNA agarose gel. The qPCR analysis of maspin mRNA expressions was performed on ABI 7500 system (Applied Biosystems, Foster, CA) by using KAPA SYBR® FAST Master Mix (2X) and was normalized to GAPDH expression. The qPCR analysis of miR-7, -103, -107, and -21 expression was performed on LightCycler 480 System (Roche, Indianapolis, IN, USA) by using KAPA Probe FAST Universal qPCR Kit and was normalized to U48 expression. Specific primers used in PCR and real-time PCR were listed in Supplemental Table S5.

Apoptosis evaluation

Cellular apoptosis induced by drug treatments was evaluated by flow cytometry (FACSCalibur Becton-Dickinson Biosciences). After drug treatments, cells were trypsinized and then fixed with 70% cold ethanol at 4°C overnight. The fixed cells were centrifuged and stained with 1 µg/mL propidium iodide and 5 µg/mL RNase at 37°C for 30 min. Analyses of 10, 000 events were carried out on a FACSCalibur, and the cell cycles were analyzed by ModFit DNA analysis software (Verity Software House). Population of subG1 indicated the drug-induced cellular apoptosis percentage.

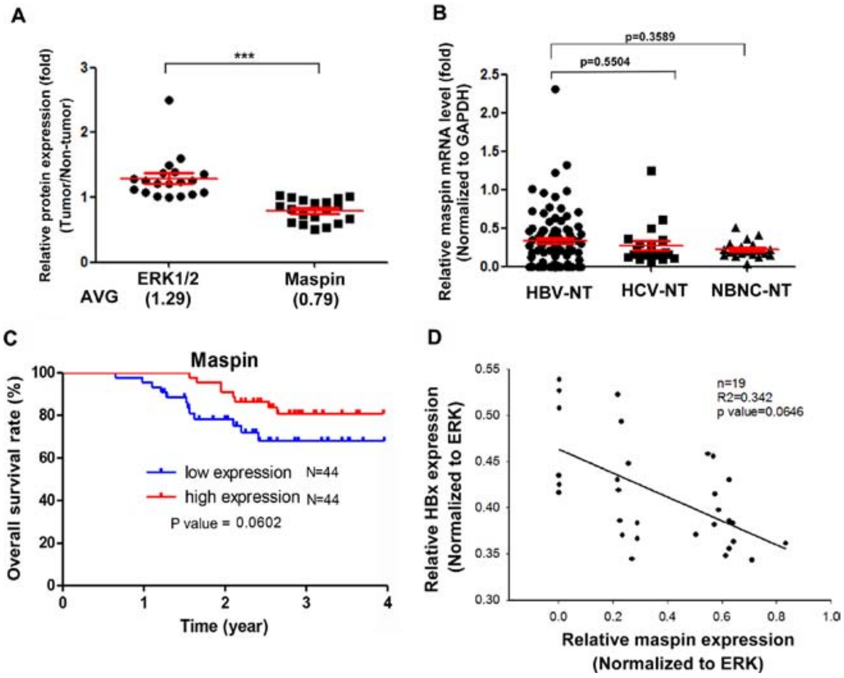

Supplementary Figure S1: Expression levels of maspin in HCC tumors. A. The total protein lysate from tumor (T) and adjacent normal tissues (N) of HBV-associated HCC tumor liver tissues were analyzed by Western blot. The protein levels of maspin were quantified with ImageJ and normalized to ERK expression level. The relative protein expression between tumor and adjacent (T/N) was analyzed by a paired Student's *t*-test. **B.** Total RNA extracted from adjacent normal tissues of HBV-, HCV-, or NBNC-associated HCC patients were analyzed by real-time qPCR with specific maspin primers. The difference in maspin expression between these normal tissues was presented as the relative expression ratio with the normalization to the mRNA level of GAPDH and was analyzed by a paired Student's *t*-test. **C.** Overall survival according to maspin mRNA expression level in HBV-associated HCC tissues was determined by Kaplan–Meier analysis. **D.** Total lysates from HBV-associated HCC tumor liver tissues were prepared and subjected to Western blot with anti-HBx, maspin, and ERK antibodies. The coefficient of determination (r^2) between HBx and maspin expression levels was analyzed by simple regression with the normalization to ERK protein level. ***$p < 0.001$.

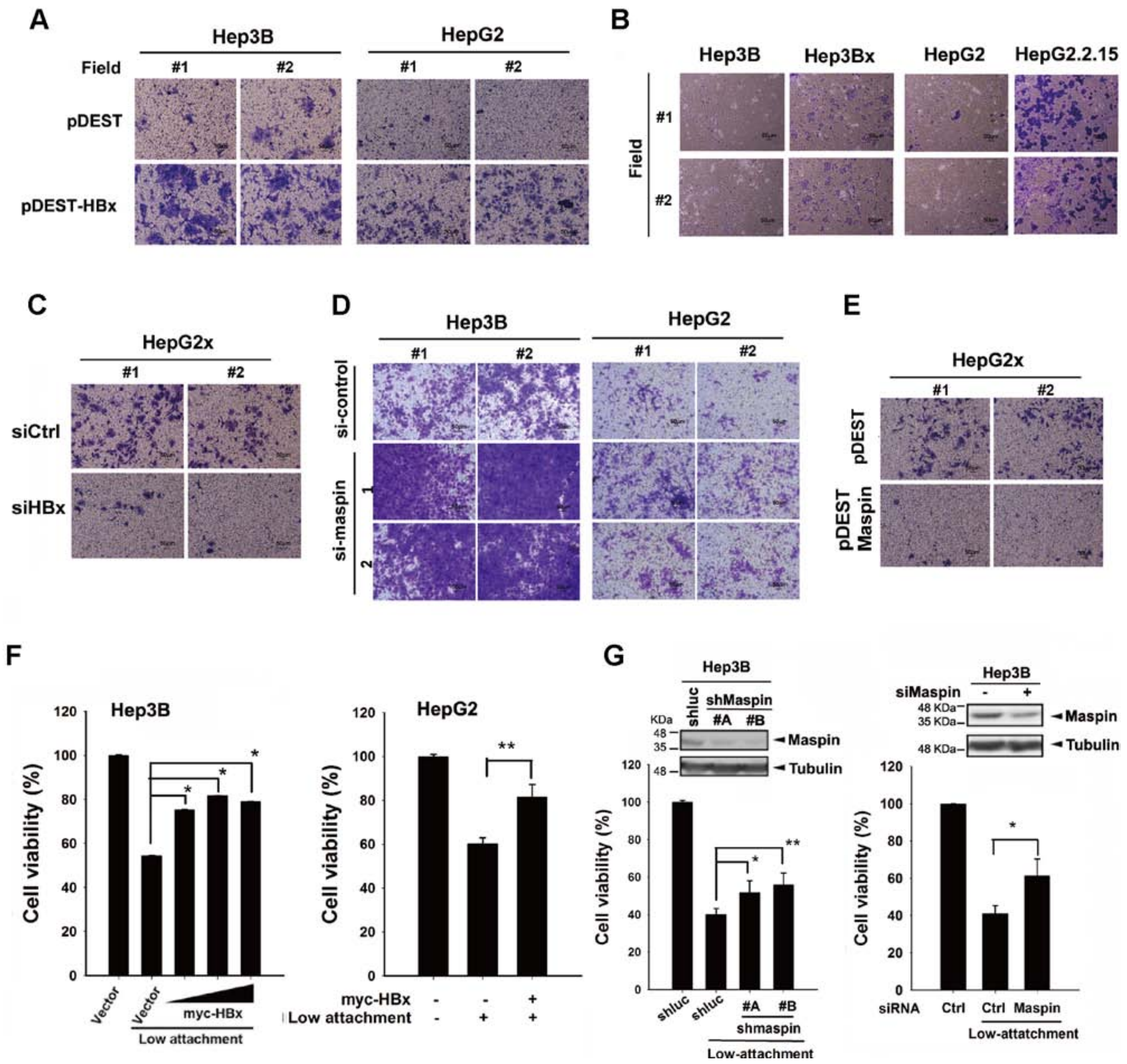

Supplementary Figure S2: Role of maspin in invasion and anoikis-induced cell death in hepatocellular carcinoma cells. A–E. The invasion capabilities of Hep3B and HepG2 cells transfected with HBx, maspin, siRNA against HBx or maspin were measured by transwell invasion assays. **F** and **G.** Hep3B cells transfected with HBx (F) and maspin shRNA or siRNA (G) were cultured on Ultra-low attachment plates followed by cell number counting assays ($n = 3$). The difference was calculated by a Student's t-test. $*p < 0.05$; $**p < 0.01$.

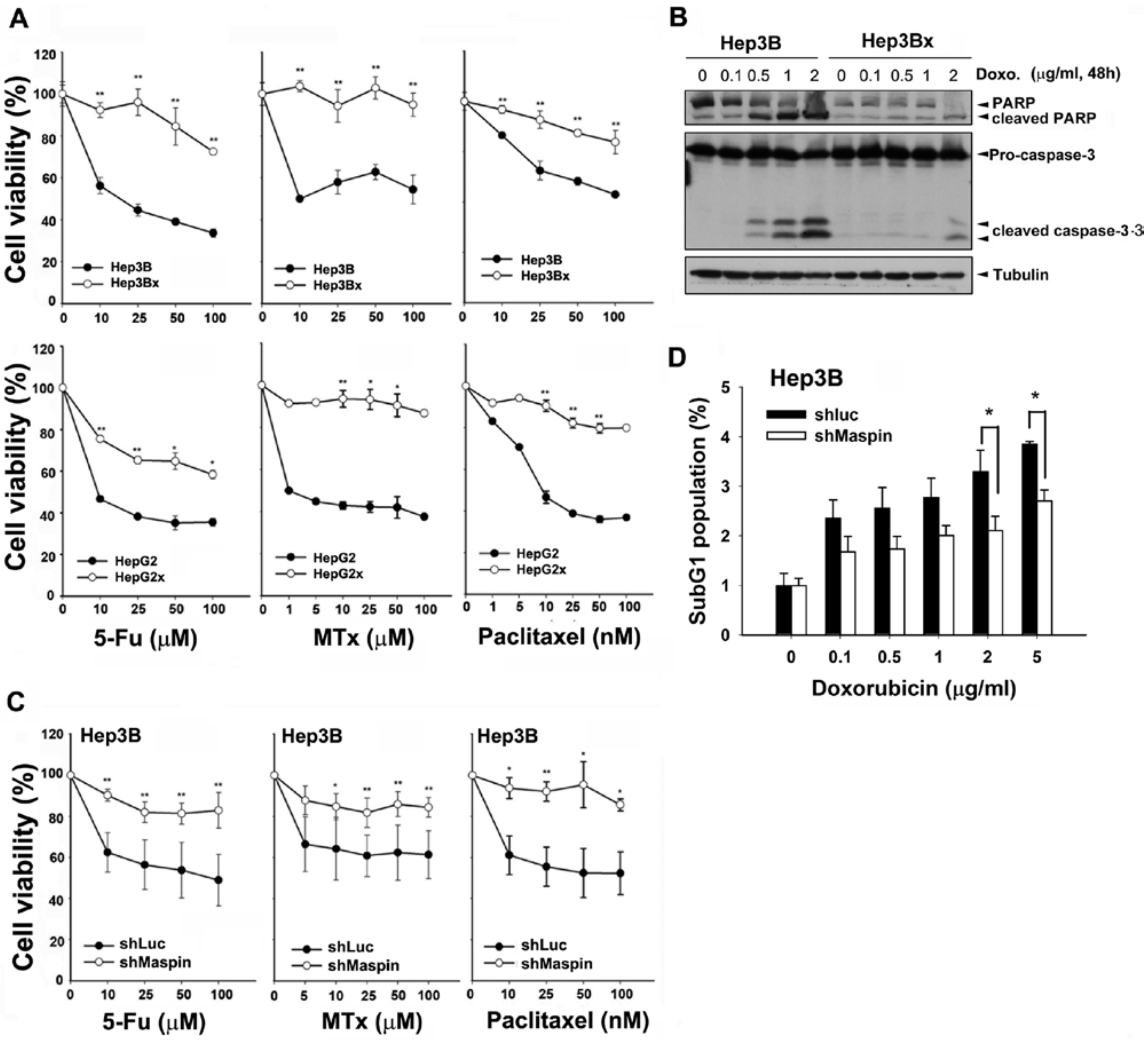

Supplementary Figure S3: Role of maspin in chemosensitivity in hepatocellular carcinoma cells. A. Hep3B/Hep3Bx and HepG2/HepG2x cells were treated with 5-Fu, MTX, and Paciltaxel for 48 hours and then were subjected to MTT assays to determine the cell viability ($n = 3$). **B.** Total protein lysates prepared from Hep3B and Hep3Bx cells treated with increasing concentration of doxorubicin for 48 hours were subjected to Western blot analysis with indicated antibodies. **C** and **D.** Maspin-knockdown Hep3B cells were treated with doxorubicin for 48 hours and subjected to MTT assays and flow cytometry ($n = 3$). The subG1 population indicated the doxorubicin-induced cell apoptosis. The difference was calculated by a Student's *t*-test. $*p < 0.05$; $**p < 0.01$.

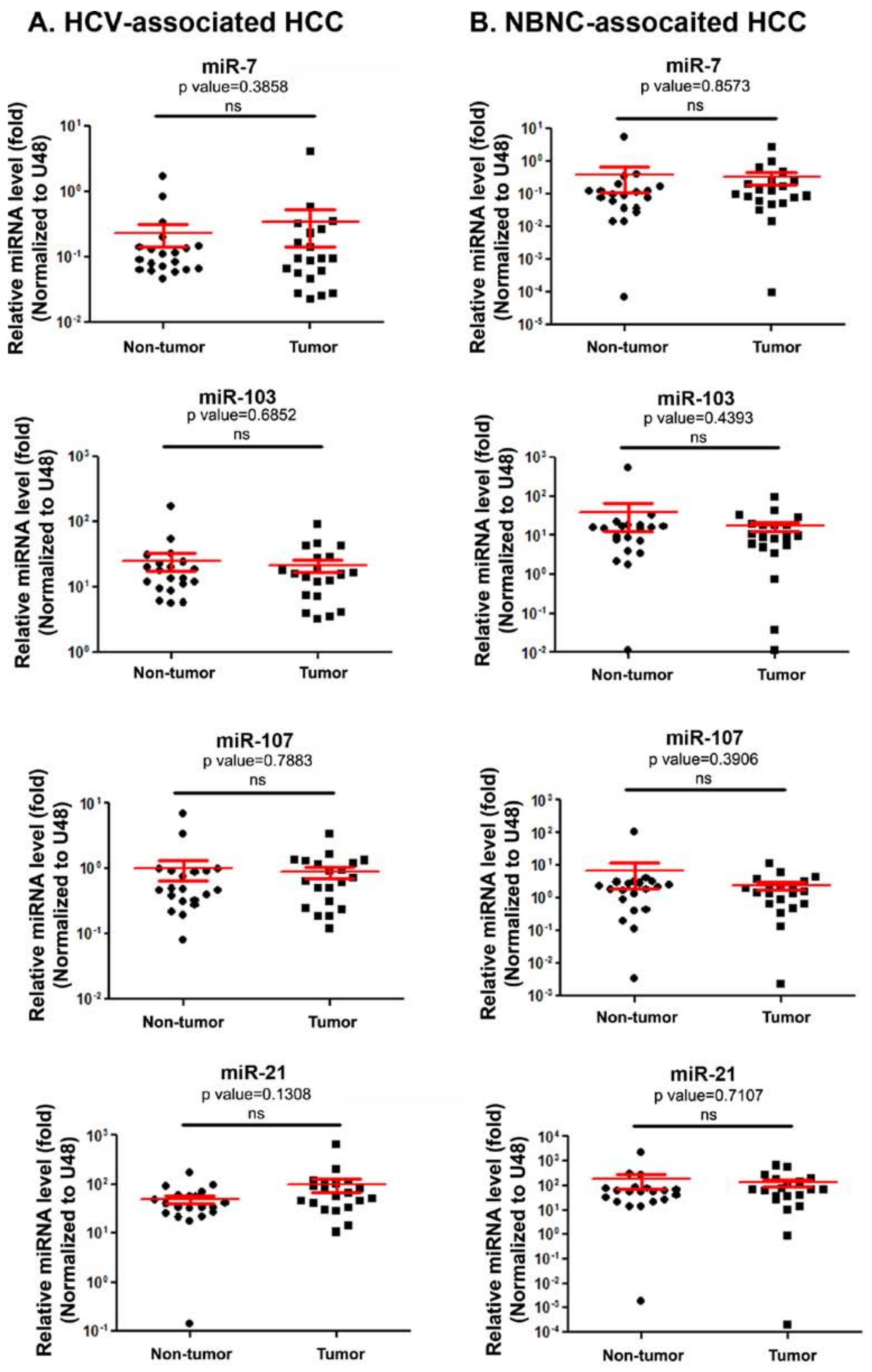

Supplementary Figure S4: Comparison of microRNA-7, -103, -107, and -21 expression in HCV- and NBNC-associated HCC tumor tissues versus their adjacent normal tissue. The total RNA extracted from tumor and adjacent normal tissues of HCV- and NBNC-associated HCC patients was analyzed by RT-qPCR for four microRNAs and U48 levels. The expression levels of individual miRNAs were normalized to U48 ($n = 20$). The differences of these microRNAs between tumor and adjacent normal tissues were calculated by a paired Student's t-test. ($^*p < 0.05$; $^{**}p < 0.01$)

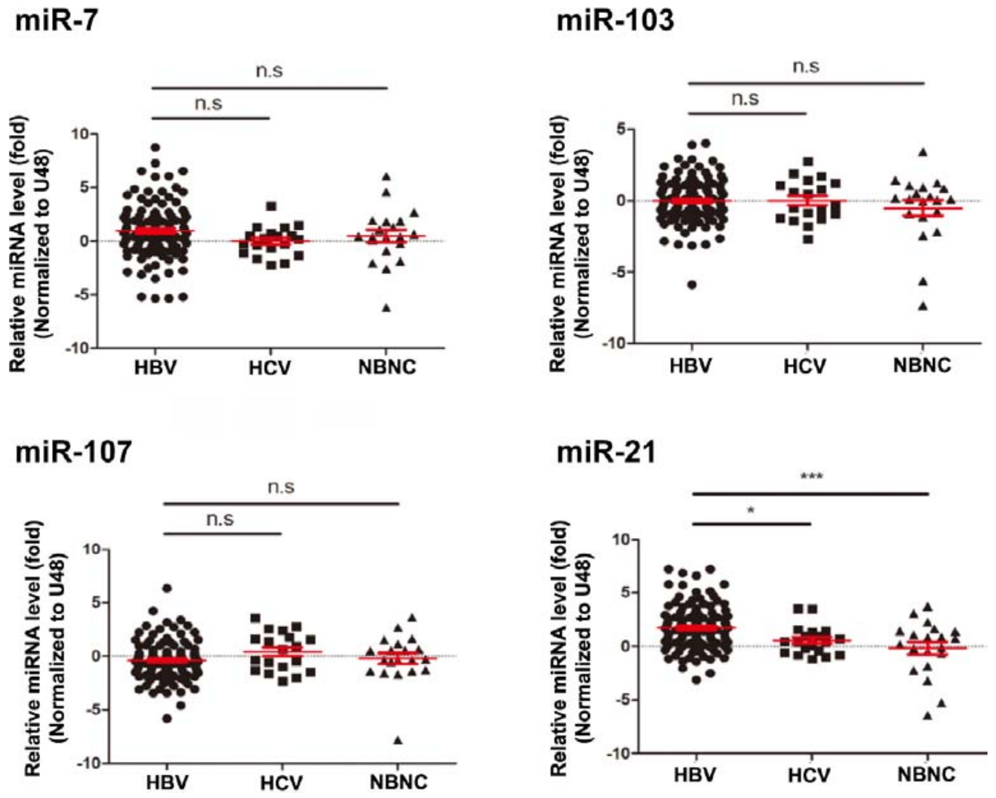

Supplementary Figure S5: Comparison of microRNA-7, -103, -107, and -21 expressions in the tumor tissues of HBV-associated HCC versus in the tumor tissue of HCV- or NBNC-associated HCC. The total RNA extracted from tumor tissues of HBV-, HCV- and NBNC-associated HCC patients was analyzed by RT-qPCR for indicated microRNAs and U48 levels. The expression levels of individual miRNAs were normalized to U48. The difference of these miRNAs level between HBV-associated and other two HCC tumors was analyzed by a Student *t*-test. (*$p < 0.05$; **$p < 0.01$; ***$p < 0.001$)

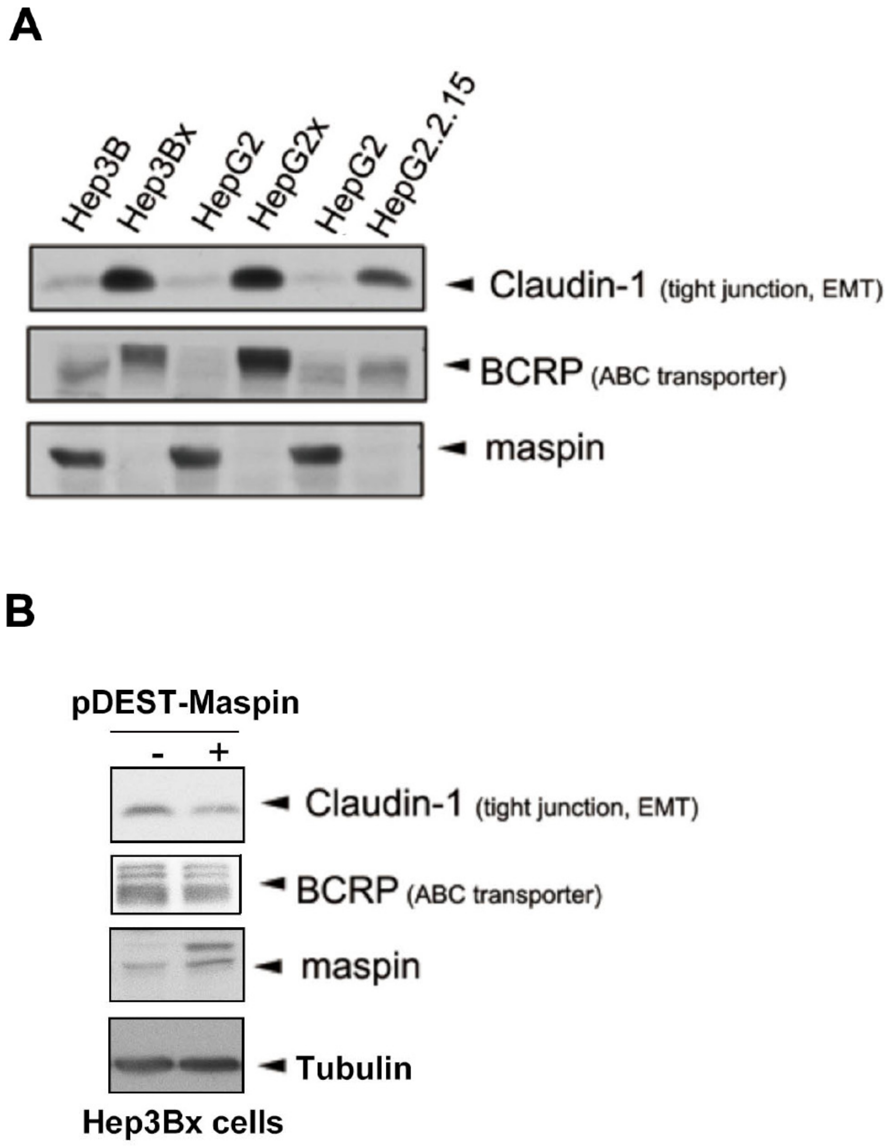

Supplementary Figure S6: Involvement maspin suppression in HBx up-regulated claudin-1 and BCRP expressions. Total protein extracted from Hep3B, Hep3Bx, HepG2, HepG2x, and HepG2.2.15 cells **A.** or from maspin-transfected Hep3Bx cells **B.** were subjected to Western blot analysis for the examination of maspin, claudin-1, and BCRP expressions.

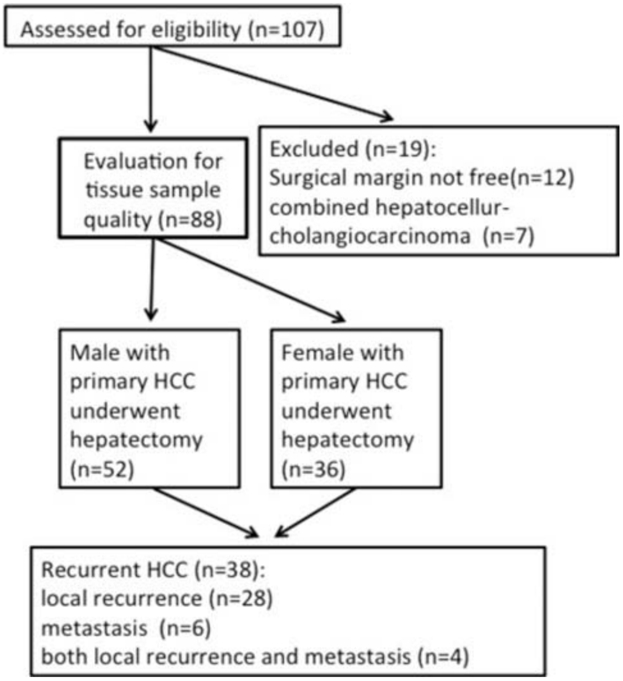

Supplementary Figure S7: The flow chart showing clinical sample information met REMARK guideline.

Supplementary Table S1. The elevated microRNAs with at least 2-fold increase in both Hep3Bx and HepG2x cells compared with their parental cells

Gene Name	
hsa_miR_93	hsa_miR_19b
hsa_miR_92	hsa_miR_194
hsa_miR_638	hsa_miR_192
hsa_miR_320	hsa_miR_191
hsa_miR_30d	hsa_miR_182
hsa_miR_29a	hsa_miR_17_5p
hsa_miR_26a	hsa_miR_16
hsa_miR_25	hsa_miR_107
hsa_miR_24	hsa_miR_106a
hsa_miR_23b	hsa_miR_103
hsa_miR_21	hsa_let_7a

The microRNA expression profiles in Hep3B, Hep3Bx, HepG2, and HepG2x were analyzed with microRNA microarray.

Supplementary Table S2. Predicted maspin-targeted microRNAs

Gene Name
hsa_miR_103
hsa_miR_107
hsa_miR_7
hsa_miR_221
hsa_miR_613
hsa_miR_1
hsa_miR_206
hsa_miR_222
hsa_miR_216a
hsa_miR_590–5p
hsa_miR_21

Prediction of maspin-targeted miRNAs was performed with three different target prediction algorithms (miRanda, Targetscan, and PITA).

Supplementary Table S3. Univariate and multivariate analysis of clinicopathologic characteristics associated with recurrence free survival in HBV-related HCC patients

Patient Characteristics	Patient numbers	Recurrence free survival			
		Univariate analysis		Multivariate analysis	
		Hazard ratio (95% CI)	*p* value	Hazard ratio (95% CI)	*p* value
Sex					
Male	53	1		1	
Female	36	0.55		0.62	
		0.24–1.34	0.386	0.38–1.21	0.76
Underlying liver disease					
Liver cirrhosis	35	1		1	
Non-cirrhosis	53	0.48		0.51	
		0.22–1.02	0.148	0.28–1.16	0.87
HCC differentiation					
Well	27	1		1	
Moderate & poor	61	1.37		1.33	
		0.89–1.86	0.234	0.74–1.68	0.33
Vascular invasion					
Yes	39	1		1	
No	49	0.34		0.64	
		0.11–0.92	0.044	0.41–1.32	0.56
AFP level					
AFP > 200 ng/ml	56	1		1	
AFP < 200 ng/ml	32	0.61		0.81	
		0.32–1.24	0.258	0.61–1.53	0.36
Pathological staging (AJCC)					
Stage I	37	1		1	
Stage II & III	51	1.58		1.38	
		1.23–1.94	0.038	0.83–1.74	0.19
Maspin level					
High expression	44	1		1	
Low expression	44	1.96		1.82	
		1.34–2.92	0.001	1.21–2.84	0.01
miR 7 level					
Low expression	44	1		1	
High expression	44	1.72		1.56	

(*Continued*)

Patient Characteristics	Patient numbers	Recurrence free survival			
		Univariate analysis		Multivariate analysis	
		Hazard ratio (95% CI)	*p* value	Hazard ratio (95% CI)	*p* value
		1.22–2.56	0.002	1.02–2.26	0.03
miR 103 level					
Low expression	44	1		1	
High expression	44	1.54		1.43	
		1.12–2.14	0.045	0.95–1.86	0.06
miR 107 level					
Low expression	44	1		1	
High expression	44	1.95		1.93	
		1.24–3.56	0.001	1.32–3.34	0.01
miR 21 level		1		1	
Low expression	44	2.67		2.47	
High expression	44	1.62–4.54	0.001	1.43–4.23	0.01

Total patients, $n = 88$

Supplementary Table S4. The basic clinical characteristic of HCV- and NBNC-associated HCC patients

Patient Characteristics	Patient numbers	
	**HCV**	**NBNC**
Sex		
Male	10	10
Female	10	10
Underlying liver disease		
Liver cirrhosis	12	4
Non-cirrhosis	8	16
HCC differentiation		
1	10	14
2	10	6
3	0	0
Vascular invasion		
Yes	8	16
No	12	4
AFP level		
AFP > 200 ng/ml	11	11
AFP < 200 ng/ml	9	9
Pathological staging (AJCC)		
Stage I	8	4
Stage II & III	12	16
Grading		
Grade 1 & 2	13	16
Grade 3 & 4	7	4
Total patients, *n* =	20	20

Supplementary Table S5. Primers used in this study

RT-qPCR primer	Sequence (5′-3′)
maspin	Forward CATGTTCATCCTACTACCCAAGG<br>Reverse TCTGAGTTGAGTTGTTTTTCAATCTT
HBx	Forward AGCGAATTCATGGCTGCTAGGCTGTGCTG<br>Reverse ACGCTCGAGTAAAGAGAGGTGCGCCCCGT
GAPDH	Forward AGCCACATCGCTCAGACAC<br>Reverse GCCCAATACGACCAAATCC
miR-7	Forward GTTGGCTCTGGTGCAGGGTCCGAGGTATTCGCACCAGAGCCAACACAACA<br>Reverse GCGGCGTGGAAGACTAGTGAT
miR-103	Forward GTTGGCTCTGGTGCAGGGTCCGAGGTATTCGCACCAGAGCCAACTCATAG<br>Reverse GCGTCCAGCAGCATTGTACAG
miR-107	Forward GTTGGCTCTGGTGCAGGGTCCGAGGTATTCGCACCAGAGCCAACTGATAG<br>Reverse GGTGGCAGCAGCATTGTACAG
miR-21	Forward GTTGGCTCTGGTGCAGGGTCCGAGGTATTCGCACCAGAGCCCAACTCAACA<br>Reverse GGCGGCTAGCTTATCAGACTG
snRNA U48	Forward GTTGGCTCTGGTGCAGGGTCCGAGGTATTCGCACCAGAGCCAACTCAGCG<br>Reverse CGGCGGTAACTCTGAGTGTGT
snRNA U6B	Forward GTTGGCTCTGGTGCAGGGTCCGAGGTATTCGCACCAGAGCCAACAAAAATAT<br>Reverse TTCCTCCGCAAGGATGACACGC
Universal Reverse primer #21	GTGCAGGGTCCGAGGT
